# Postoperative outcomes of ligasure use during common femoral artery exposure

**DOI:** 10.1016/j.jvscit.2026.102248

**Published:** 2026-04-09

**Authors:** Koyal Ansingkar, Brittany Landavazo, Brianna Elys Whithorn, Maham Rahimi

**Affiliations:** a School of Engineering Medicine, Texas A&M University, Houston, TX; b College of Medicine, Texas A&M University, Round Rock, TX; c DeBakey Heart & Vascular Center, Houston Methodist Hospital, Houston, TX

**Keywords:** Femoral artery, Postoperative complications, Lymphatic system injuries, Seroma, Electrosurgery

## Abstract

**Objective:**

Seroma formation after common femoral artery (CFA) exposure is a recognized complication in vascular surgery, associated with delayed wound healing and graft-related morbidity. Reported seroma rates following CFA exposure using standard electrocautery range from 5% to 15% for cases requiring operative reintervention. The LigaSure bipolar vessel sealing system has demonstrated efficacy in sealing lymphatic channels in other surgical specialties, but its safety and impact in vascular groin surgery have not been well-characterized. We evaluated the safety, feasibility, and postoperative outcomes associated with LigaSure use during CFA exposure.

**Methods:**

We conducted a retrospective cohort study of consecutive patients undergoing open CFA exposure using LigaSure at a tertiary vascular referral center between June 2023 and November 2025, excluding any trauma cases. Demographic, clinical, operative, and postoperative outcome data were abstracted from the electronic medical record. The primary outcome was postoperative seroma formation, with clinical significance defined as the need for procedural or operative reintervention. Secondary outcomes included hematoma, surgical site infection, and fat necrosis. Observed outcome rates were descriptively compared with historically reported seroma rates after femoral artery exposure. Postoperative follow-up occurred during the index hospitalization and at scheduled outpatient visits.

**Results:**

Twenty-eight patients were included. Cardiovascular comorbidities were prevalent: hypertension (86%), hyperlipidemia (75%), and diabetes (50%). Four patients (14.3%; 95% confidence interval [CI], 4.03%-32.67%) developed postoperative fluid collections: two seromas (7.1%; 95% CI, 0.88%-23.50%) and two hematomas (7.1%; 95% CI, 0.88%-23.50%), comparable with historically reported seroma rates of 5% to 15% after standard electrocautery. Critically, no patients required procedural drainage, operative reintervention, or graft excision, and no graft infections or fat necrosis were observed. Only one case of skin infection and one case of skin necrosis were noted. All fluid collections resolved with conservative management, yielding a final rate of 0% for seroma requiring operative reintervention.

**Conclusions:**

LigaSure use during CFA exposure was safe and feasible, with postoperative seroma rates comparable with those historically reported for standard electrocautery. Notably, 0% of seromas required procedural or operative reintervention, an important clinical consideration given the morbidity often associated with groin seromas in vascular surgery. Although a decrease in seroma incidence was not observed relative to historical benchmarks, the favorable safety profile and absence of clinically significant lymphatic complications requiring operative reintervention support LigaSure as a reliable adjunct for groin dissection. Larger prospective comparative studies are needed to further define its impact on clinically meaningful groin wound outcomes.

**Article Highlights**

• **Type of Research:** Single-center retrospective cohort study
• **Key Findings:** In 28 patients that underwent common femoral artery exposure with LigaSure, postoperative fluid collections occurred in 4 cases (2 seromas, 2 hematomas), all of which resolved with conservative management. No patients required procedural drainage, operative reintervention, or graft excision, and no graft infections or fat necrosis was observed.
• **Take Home Message:** LigaSure use during femoral artery exposure appears safe and feasible, with no clinically significant seromas requiring intervention, supporting its use as a reliable adjunct for vascular groin dissection.

Seroma formation remains a clinically important complication following common femoral artery (CFA) exposure in vascular surgery and is associated with increased morbidity, including delayed wound healing, surgical site infection (SSI), and prolonged hospitalization.^1^^,^^2^ Groin wound complications after vascular procedures have been consistently linked to adverse clinical outcomes, increased reintervention rates, and higher health care utilization.^3^ Importantly, the clinical burden of seroma is driven not merely by its incidence, but by the subset of cases that require procedural drainage or operative reintervention.^4^ Although predictive models have been developed to identify patients at high risk for groin complications, optimal preventative strategies to reduce clinically significant seroma formation remain controversial.^5^

The contemporary vascular literature demonstrates that postoperative groin complications remain common after femoral artery exposure, with reported seroma rates ranging from 5% to 15%, depending on patient comorbidities, the extent of lymphatic disruption, and operative technique.^2^^,^^5^^,^^6^ Notably, these reported rates largely reflect seromas that ultimately require procedural management or reoperation. A study by Dang et al^6^ showed that in 1 multicenter cohort of 136 groin incisions, postoperative fluid collections, including seroma and hematoma, occurred in a substantial proportion of patients undergoing femoral exposure, with a notable subset requiring procedural intervention or reoperation. Groin wound complications in this cohort were also associated with increased length of stay and higher rates of downstream morbidity.^6^ Reported rates of seroma formation in these series closely parallel rates of procedural intervention, underscoring the clinical impact of postoperative fluid collections.

Lymphatic injury during groin dissection, particularly in the setting of redo operations, scarred tissue planes, or prominent femoral lymphatics, plays a central role in postoperative fluid accumulation.^7^ Beyond patient discomfort, seromas are associated with delayed wound healing and may serve as a nidus for bacterial growth, thereby increasing the risk of SSI.^5^ Persistent or infected seromas may necessitate repeated aspirations, prolonged antibiotic therapy, or operative reintervention, contributing to extended hospital stays and increased costs of care.^8^

The clinical significance of postoperative fluid collections is further supported by outcome data. In a retrospective cohort study of patients undergoing lower extremity revascularization with groin incisions, 22% developed a postoperative seroma or hematoma, and 31% developed an SSI. Importantly, postoperative seroma or hematoma was identified as an independent predictor of SSI, with an odds ratio of 27.6 (95% confidence interval [CI], 5.4-139.6). Patients who developed SSI experienced longer hospital stays (14.5 days vs 8.7 days) and higher reoperation rates (49% vs 4%) compared with those without infection.^9^ These findings underscore the importance of strategies aimed at minimizing lymphatic disruption during femoral artery exposure to decrease downstream morbidity.

Although traditional monopolar electrocautery is effective for hemostasis, monopolar electrocautery is associated with greater lateral thermal spread and tissue injury, which may result in only transient lymphatic sealing and predispose to postoperative seroma formation.^10^ In contrast, the LigaSure (Medtronic) bipolar vessel sealing system offers controlled thermal spread, consistent sealing of vessels ≤7 mm, as well as feedback-regulated energy delivery, features that may theoretically improve lymphatic management. The average seal cycle is between 2 and 4 seconds when used with the ForceTriad energy platform, and the seals can withstand three times normal systolic blood pressure. Through feedback-controlled response technology, LigaSure automatically discontinues thermal energy when the cycle is complete, reducing the amount of subjective judgment in determining hemostasis.^11^ The reduced dependence on operator judgment and minimized thermal spread may offer advantages in anatomically confined and lymphatic-rich operative fields, such as the femoral triangle. LigaSure is commonly used in colorectal, gynecological, urological, and general laparoscopic surgery owing to its efficiency in vessel sealing and effective hemostasis critical to surgical success and patient safety.

Given the close alignment between reported seroma rates and the need for procedural intervention in prior series, strategies that safely limit these clinically significant postoperative fluid collections are of particular interest in vascular groin surgery. Despite widespread adoption of bipolar vessel sealing devices in other lymphatic-rich operative fields, their use during femoral artery exposure has not been well-characterized. Only one prior study evaluating electrofusion technology in femoral exposure demonstrated reduced lymphatic complications, but LigaSure specific data are lacking.^12^ The present study examines postoperative outcomes after CFA exposure performed with the LigaSure bipolar vessel sealing system, with particular attention to seroma formation, the need for procedural or operative reintervention, and wound-related complications.

## Methods

### Study design and patient selection

This study was a retrospective cohort analysis conducted at a single tertiary vascular referral center following approval by the Houston Methodist Research Institute Institutional Review Board (Protocol PRO00024971:1), with a waiver of informed consent. Patients who underwent open femoral artery exposure between June 2023 and November 2025 were eligible for inclusion if LigaSure (Medtronic) was used as the primary dissecting and vessel-sealing device during the groin incision and femoral exposure. Patients undergoing femoral exposure for trauma were excluded. For patients who underwent bilateral femoral exposure, outcomes were recorded at the patient level rather than separately for each groin. One patient underwent a redo operation using LigaSure for excision of an infected graft, which was placed at an outside hospital 6 days prior.

Postoperative outcomes in the current cohort were descriptively contextualized against seroma and intervention rates previously reported in our institution's published vascular surgery series evaluating CFA exposure performed with standard electrocautery, in which the incidence of seroma requiring operative reintervention ranged from 5% to 15%. These historical data were used for contextual benchmarking rather than formal statistical comparison.

### Data collection and variables

Demographic data collected included age, sex, race, ethnicity, and body mass index. Clinical variables included the presence of hypertension, hyperlipidemia, diabetes mellitus, chronic kidney disease or end-stage renal disease, and tobacco use history. Operative characteristics abstracted from the medical record included surgical indication, incision type, use of prosthetic graft material, operative time, estimated blood loss, and perioperative blood transfusion requirements.

### Outcome definitions

Postoperative outcomes were assessed through a review of inpatient and outpatient records and included the development of groin fluid collections (seroma or hematoma), SSI, graft infection, fat necrosis, need for reintervention, and documented findings on follow-up examination or imaging. For analytic purposes, particular emphasis was placed on clinically significant fluid collections, defined as those requiring percutaneous drainage or operative reintervention.

Seroma was defined as a clinically evident or radiographically identified postoperative fluid collection within the groin, not consistent with hematoma, which was defined as a collection associated with acute postoperative bleeding. Seromas were further categorized by management strategy to distinguish seromas that spontaneously resolved from clinically consequential seromas.

Fluid collections were identified during routine postoperative clinical follow-up and through review of imaging studies obtained for symptomatic patients or concerning physical examination findings; routine postoperative imaging was not performed. Patients were followed for postoperative wound-related complications through the initial postoperative visit, typically within 30 days of surgery. Medical records for these patients were reviewed for the 6 months after their index procedure, to encompass any additional findings. Imaging studies were obtained at the discretion of the treating surgeon for symptoms or physical examination findings.

### Statistical analysis

Given the exploratory nature of the study, the small sample size, and the absence of a comparator cohort, analyses were limited to descriptive statistics. Continuous variables are reported as mean ± standard deviation, and categorical variables are presented as counts and percentages. Observed rates of postoperative seroma and fluid collections were contextualized against historical benchmarks reported in the literature rather than subjected to inferential statistical comparison. We calculated 95% CIs for rates using the Clopper-Pearson exact method. No inferential or multivariable statistical analyses were performed.

## Results

### Patient characteristics

Twenty-eight patients underwent femoral artery exposure with LigaSure during the study period. The mean age was 63 ± 14 years, and 15 patients (53.57%) were female. Cardiovascular comorbidities were prevalent throughout the cohort, including hypertension in 24 patients (85.7%), hyperlipidemia in 21 (75%), and diabetes mellitus in 14 (50%). Complete demographic and clinical characteristics are summarized in Table I. Mean clinical follow-up was 30.14 ± 39.62 days, with 13 patients (46.43%) followed beyond 30 days postoperatively. One patient underwent a redo operation using LigaSure for excision of an infected graft, which was placed at an outside hospital.

**Table I:** Demographic and clinical data for patients who underwent common femoral artery (CFA) exposure procedures using LigaSure

Characteristic	Value
Age, years	63 ± 14
Female sex	15 (53.57)
Body mass index, kg/m^2^	28.05 ± 6.85
Hypertension	24 (85.7)
Hyperlipidemia	21 (75.0)
Type 2 diabetes mellitus	14 (50.0)
Chronic kidney disease or end-stage renal disease	11 (39.29)
Tobacco use history	5 current (17.86)
	14 former (50.0)
Redo femoral exposure	1 (3.57)

Values are mean ± standard deviation or number (%).

### Operative characteristics and technique

Procedures performed spanned a range of vascular operations, including femoral endarterectomy, pseudoaneurysm repair, femoropopliteal bypass, aortobifemoral bypass, extracorporeal membrane oxygenation decannulation, and thrombectomy (Table II). The skin incisions were made with standard scalpels. LigaSure was used consistently for lymphatic and soft tissue dissection during femoral exposure in all cases. No electrocautery was used, and free ties were only used for any observed vessels >7 mm in diameter that could not be sealed with LigaSure. Device use followed standard manufacturer settings per surgeon discretion. Closed-suction drains were placed at the discretion of the operating surgeon in three patients (10.71%). Each patient underwent negative wound pressure therapy with Prevena (Solventum).

**Table II:** Characteristics of the surgical intervention each patient with a postoperative fluid collection underwent

Variable	Value
Procedure type	
Pseudoaneurysm repair	5 (17.86)
Lower extremity bypass	6 (21.43)
Aortobifemoral bypass	3 (10.71)
ECMO decannulation	5 (17.86)
Thrombectomy	3 (10.71)
Other	6 (21.43)
Prosthetic graft used	14 (50.00)
Operative time, minutes	216.58 ± 131.74
Estimated blood loss, mL	951.23 ± 3286.61
Perioperative transfusion	12 (42.86)
Closed-suction drain placed	3 (10.71)
Negative pressure wound vac therapy	28 (100)

*ECMO*, Extracorporeal membrane oxygenation.

Values are mean ± standard deviation or number (%).

### Postoperative fluid collections

No patient developed a postoperative groin fluid collection requiring percutaneous drainage, operative reintervention, or prolonged hospitalization, yielding a 0% rate of clinically significant seroma.

Overall, postoperative groin fluid collections occurred in 4 patients (14.3%; 95% CI, 4.03%-32.67%), including two seromas (7.1%; 95% CI, 0.88%-23.50%) and two hematomas (7.1%; 95% CI, 0.88%-23.50%). Both seromas occurred in patients undergoing prosthetic bypass procedures (2 of 14 prosthetic graft cases). Neither patient had a closed suction drain placed at the time of operation. One patient with seroma underwent a longitudinal incision, and the other underwent an oblique incision. Neither seroma was associated with clinical signs of infection, leukocytosis beyond expected postoperative values, or hemodynamic instability. One seroma was evaluated with cross-sectional imaging and measured 2.1 × 1.0 cm, and the other was identified clinically without imaging confirmation. All fluid collections resolved within 2 weeks of surgery.

One patient developed a superficial skin infection requiring incision and drainage, and one patient developed localized skin necrosis requiring excisional debridement. Neither complication occurred in patients with seroma, nor were there graft infections or cases of fat necrosis observed during the follow-up period. Notably, one patient underwent a redo groin incision for excision of an infected graft; this patient experienced no postoperative seroma, hematoma, or other wound-related complications. These findings are summarized in Table III.

**Table III:** Postoperative wound outcomes

Outcome	No. (%)
Any fluid collection	4 (14.3)
Seroma	2 (7.1)
Hematoma	2 (7.1)
SSI	1 (3.6)
Skin necrosis	1 (3.6)
Graft infection	0
Fat necrosis	0
Reintervention for seroma	0

*SSI*, Surgical site infection.

## Discussion

This study represents one of the first vascular-specific evaluations of LigaSure for femoral artery exposure. Postoperative seroma occurred in 2 of 28 patients (7.1%), consistent with previously reported rates for CFA exposure using standard electrocautery (5%-15%).^2^ Importantly, no patients required reintervention or drainage, no graft infections or fat necrosis were observed, and no patients experienced any femoral nerve injuries or cutaneous skin trauma, suggesting that LigaSure use is safe in this context.

### Comparison to existing literature

Prior studies have explored strategies to reduce seroma formation, with lymphatic ligation remaining as the gold standard in vascular and other surgical fields.^13^, ^14^, ^15^ In other specialties such as breast and melanoma surgery, LigaSure has demonstrated reductions in drain output and lymphatic leakage.^16^ However, the pathophysiology of lymphatic injury in CFA exposure differs from full lymph node basin dissections, which may explain the more modest findings in our cohort. During lymph node dissection, the risk of seroma formation is inherently higher owing to manipulation and excision of the lymph channels. Thus, devices that optimize long-term sealing of these vessels may have a more robust impact on true lymph node dissection.^17^^,^^18^

In CFA exposure, manipulation of the lymphatic vessels in the groin is theorized to be the cause of postoperative seroma. However, lymph node dissection is not required in CFA exposure, which may account for findings of seroma formation at a rate comparable to existing literature. Although electrocautery remains widely used, concerns about thermal injury, partial lymphatic sealing, and fat necrosis drive interest in alternative devices.^10^^,^^14^ The more controlled thermal profile of bipolar vessel-sealing systems offers a theoretical advantage; in our cohort, LigaSure was associated with zero thermal injuries or fat necrosis, and no clinically significant seromas occurred that required operative or procedural intervention.

### Clinical implications

CFA exposure carries inherent risk for seroma formation owing to proximity to lymphatic channels, deep dissection planes, and dead space creation. In vascular surgery, postoperative groin fluid collections are of particular concern because they may compromise prosthetic grafts and frequently necessitate procedural intervention, contributing to increased morbidity.^8^ As described, contemporary vascular series evaluating femoral artery exposure demonstrated that postoperative groin fluid collections frequently translate into clinically significant interventions. In the study by Dang et al,^6^ rates of postoperative seroma and hematoma closely paralleled rates of procedural drainage and return to the operating room, underscoring that fluid collections identified in clinical practice often represent events requiring intervention rather than incidental findings.

In contrast, in our cohort, no postoperative fluid collections required drainage or reoperation, emphasizing that clinically significant seromas were absent despite a 7.1% overall seroma rate. This finding highlights that LigaSure use during CFA exposure does not increase the risk of clinically consequential seroma formation when benchmarked against reported historical experience. Two patients experienced skin complications (one superficial infection, one localized necrosis), neither of which involved seroma or prosthetic grafts, and both were managed successfully without affecting the primary outcome. The single patient in this cohort who underwent a redo groin incision for excision of an infected AV graft experienced no postoperative complications, suggesting that LigaSure may maintain safety even in higher-risk or reoperative groin dissections.

Comparisons with historical seroma rates should be interpreted with caution, because definitions of seroma, imaging protocols, and postoperative surveillance vary widely across studies.^15^ Differences in clinical vs imaging-confirmed seroma and follow-up intensity may contribute to variability in reported incidence. Nevertheless, the observed safety profile, including the absence of graft infection, fat necrosis, or reoperation, supports LigaSure as a feasible and safe tool for groin dissection, with potential to minimize clinically significant lymphatic complications.

### Study limitations

Limitations of this study include small sample size, limiting statistical inference. The single-arm design of the study precludes direct comparison with electrocautery. Procedures included a range of vascular operations, including bypass, pseudoaneurysm repair, thrombectomy, and extracorporeal membrane oxygenation decannulation. Differences in operative complexity and lymphatic manipulation may influence seroma risk and limit generalizability. Last, the retrospective design of the study may pose a lack of knowledge of seromas diagnosed outside of the follow-up period. Despite these limitations, this study contributes meaningful vascular specific data to an area with limited evidence.

### Future directions

Although this study suggests potential benefits of electrofusion-based systems in reducing serous output, the role of LigaSure in vascular procedures remains insufficiently characterized and warrants further investigation. Future investigation should focus on defining the comparative effectiveness of LigaSure vs standard electrocautery during CFA exposure using clinically meaningful end points. A prospective, randomized study with standardized operative technique and postoperative surveillance would allow for direct comparison of seroma formation, need for procedural drainage, reoperation, and graft-related infection. Given the disproportionate clinical impact of groin complications in vascular surgery, future trials should prioritize intervention-requiring fluid collections rather than imaging-only findings. Stratification by procedure type, prosthetic graft use, and adjunctive wound management strategies may further clarify patient and operative factors that influence lymphatic outcomes after femoral exposure. Adjunctive strategies (lymphatic ligation, dead space obliteration, and negative pressure dressings) should also be integrated into future trial designs.

## Conclusion

LigaSure use during femoral artery exposure was safe and feasible, with no observed graft infections, reinterventions, or fat necrosis in this cohort. Although the overall incidence of postoperative seroma was comparable to rates historically reported for standard electrocautery, none of the fluid collections in this cohort required procedural drainage or operative intervention, highlighting the absence of clinically significant seromas.

These findings support the vascular-specific safety of bipolar vessel sealing technology and highlight the importance of evaluating intervention-based outcomes, rather than seroma incidence alone, when assessing strategies to optimize groin wound management. Larger prospective, comparative studies with standardized definitions and surveillance protocols are needed to more definitively determine whether LigaSure influences the severity, clinical course, or management of postoperative groin seromas.

## Author Contributions

Conception and design: KA, BL, MR

Analysis and interpretation: KA, BL, BW, MR

Data collection: KA, BW, MR

Writing the article: KA, BL, MR

Critical revision of the article: KA, BL, BW, MR

Final approval of the article: KA, BL, BW, MR

Statistical analysis: Not applicable

Obtained funding: Not applicable

Overall responsibility: MR

## Funding

None.

## Declaration of generative AI and AI-assisted technologies in the writing process

During the preparation of this work the authors used ChatGPT in order to enhance clarity and readability. After using this tool/service, the authors reviewed and edited the content as needed and take full responsibility for the content of the publication.

## Disclosures

None.
